# *Plasmodium vivax Pv*12 B-cell epitopes and HLA-DRβ1*-dependent T-cell epitopes *in vitro* antigenicity

**DOI:** 10.1371/journal.pone.0203715

**Published:** 2018-09-10

**Authors:** Yoelis Yepes-Pérez, Carolina López, Carlos Fernando Suárez, Manuel Alfonso Patarroyo

**Affiliations:** 1 Molecular Biology and Immunology Department, Fundación Instituto de Immunología de Colombia (FIDIC), Bogotá D.C., Colombia; 2 MSc Programme in Microbiology, Universidad Nacional de Colombia, Bogotá D.C., Colombia; 3 PhD Programme in Biomedical and Biological Sciences, Universidad del Rosario, Bogotá D.C., Colombia; 4 Bio-mathematics Department, Fundación Instituto de Inmunología de Colombia (FIDIC), Bogotá D.C., Colombia; 5 Universidad de Ciencias Aplicadas y Ambientales (U.D.C.A), Bogotá D.C., Colombia; 6 Basic Sciences Department, School of Medicine and Health Sciences, Universidad del Rosario, Bogotá D.C., Colombia; Central University of Tamil Nadu, INDIA

## Abstract

Malaria is an infectious disease caused by parasites from the genus *Plasmodium (P*. *falciparum* and *P*. *vivax* are responsible for 90% of all clinical cases); it is widely distributed throughout the world’s tropical and subtropical regions. The *P*. *vivax Pv*12 protein is involved in invasion, is expressed on merozoite surface and has been recognised by antibodies from individuals exposed to the disease. In this study, B- and T-cell epitopes from *Pv*12 were predicted and characterised to advance in the design of a peptide-based vaccine against malaria.

For evaluating the humoral response of individuals exposed to natural *P*. *vivax* infection from two endemic areas in Colombia, BepiPred-1.0 software was used for selecting B-cell epitopes. B-cell epitope 39038 displayed the greatest recognition by naturally-acquired antibodies and induced an IgG2/IgG4 response. NetMHCIIpan-3.1 prediction software was used for selecting peptides having high affinity binding for HLA-DRβ1* allele lineages and this was confirmed by *in-vitro* binding assays. T-epitopes 39113 and 39117 triggered a memory T-cell response (Stimulation Index≥2) and significant cytokine production.

Combining *in-silico*, *in-vitro* and functional assays, two *Pv*12 protein regions (containing peptides 39038, 39040, 39113 and 39117) have thus been characterised as promising vaccine candidates against *P*. *vivax* malaria.

## Introduction

Malaria caused by *Plasmodium vivax* is an important public health problem in many countries worldwide; 13.8 million cases occurred in 2015, causing about 50% of all cases of malaria outside of Africa [1]. According to the PAHO/WHO Epidemiological Alert for malaria, between 2015 and 2016, an increase in malaria cases caused by *P*. *vivax* have occurred in the Americas, specially Colombia [2]. Although vector control, antimalarial drug therapy and case management have reduced the amount of events, vaccine development is considered one of the most promising strategies for controlling malaria [1]. Vaccine development strategies include immunisation with irradiated sporozoites (Spz) [3], inducing a protective response with knock-out parasites [4], identifying and expressing proteins which are important in parasite invasion to targeted hosts and searching for immune response-inducing peptides [5].

Given the lack of availability of an *in vitro P*. *vivax* culture, the development of an effective vaccine has been delayed (compared to progress in the *P*. *falciparum* field) [6]. However, studying homologous *P*. *falciparum* proteins expressed during blood-stage has led to potential *P*. *vivax* vaccine candidates being recognised and characterised [7–12]. Our group has been using a vaccine development strategy based on characterising ~20 amino acid (aa) long conserved peptides involved in target cell invasion and later modifying them to improve their interaction with major histocompatibility complex (MHC) class II molecules, such peptides have been called modified high binding activity peptides (mHABPs). This approach is the basis for developing anti-*P*. *falciparum* chemically-synthesised vaccines, based on protein subunits from the parasite’s development stages (i.e. multi-antigen, multistage) [13].

The use of these peptides as potential vaccine candidates will depend on their ability to act as efficient epitopes that could trigger the cellular immune response mediated by T cells, and/or the humoral response, mediated by B cells. Using algorithms for determining the potential T- and B-cell epitopes helps to save time and resources required for lab experiments of the pathogen’s proteins. Such tools have been here used to select *Pv*12-based synthetic peptides as potential vaccines candidates against *P*. *vivax*.

Previous studies have shown that an immune response against malaria parasites is strongly associated with these peptides’ specific binding to MHC DR class II (MHC-DR or HLA-DR (human leukocyte antigen in humans)) [13,14]. This cell surface receptor consists of a protein complex formed by HLA-DRα and HLA-DRβ having high polymorphism in humans, focusing on HLA-DRβ whilst HLA-DRα is virtually monomorphic; for sake of simplicity the complex is referred to as HLA-DRβ hereafter. Each HLA-DR molecule has differences regarding antigen binding affinity, suggesting that peptide-MHC complex formation depends on allele lineage [13,15,16].

Bioinformatics tools have been developed for predicting T-cell epitopes according to their HLA-DR allele binding profile [17–21]. Identifying high-binding HLA-DR peptides (promiscuous or allele-specific) able to trigger an effective immune response forms part of the development of a vaccine covering much of the exposed population’s HLA polymorphism [22–24].

Antigen-antibody interaction is the main characteristic of a humoral immune response; however, antibodies only recognise a portion of an antigen (known as antigenic determinant or epitope) [25,26]. Some vaccine development strategies involve using bioinformatics tools for identifying B-cell epitopes in protein vaccine candidates. Mapping linear B-cell epitopes (continuous aa in a protein sequence) through *in silico* methods involves using predictors for analysing a combination of aa physico-chemical properties, such as solvent accessibility, hydrophilicity and flexibility [27,28].

*P*. *vivax* proteomic profile studies have led to identifying vaccine candidate proteins involved in parasite invasion [5,9,29]; *Pv*12 is one of such proteins. It consists of 362 aa, having a ~41 kDa molecular mass, an N-terminal signal peptide (SP), a glycosylphosphatidylinositol (GPI) anchor in the C-terminal transmembrane domain (TM) and two 6-Cys domains [30,31]. Although initially characterised as a rhoptry protein [30], later studies confirmed its location on merozoite (Mrz) surface, like *Pf*12 [31,32]. It has been recognised by 49% of sera from individuals with active infection by *P*. *vivax*’s protein array [30]; another study revealed 84.8% of sera having an antibody response via protein recognition by ELISA (Enzyme-Linked Immunosorbent Assay) [31].

It has been shown that several 6-Cys family members play a role in ligand binding to other proteins, suggesting that *Pv*12 could play an active role in interacting with other cell receptors [31]. Genetic studies have shown that the *Pv*12 gene is highly conserved amongst *Plasmodium* species, having low genetic diversity between isolates and being one of the most conserved antigens encoding genes characterised to date for *P*. *vivax*. *Pv*12 may thus be a promising candidate for developing a vaccine against this parasite [33].

The present study used bioinformatics tools for selecting *Pv*12 B- and T-cell epitopes and evaluating their *in vitro* antigenicity. The humoral response to *Pv*12 was assessed by ELISA using sera from people living in two *P*. *vivax* endemic areas in Colombia for identifying IgG (Immunoglobulin G) and subclasses against B-epitopes. T-cell epitopes were evaluated by *in vitro* binding assays to HLA-DRβ1*0401, 1*0701, 1*1101 and 1*1302 molecules; lymphoproliferation and cytokine production assays were then carried out for those peptides displaying high binding affinity, using peripheral blood mononuclear cells (PBMC), according to their HLA-DR profile. Overall, this work has shown that the use of bioinformatics techniques combined with functional experiments yielded fruitful results, offering two regions combining B- and T-cell epitopes from *Pv*12, that can be used as components of a vaccine against *P*. *vivax*.

## Materials and methods

### Study area

Peripheral blood (PB) samples were taken from *P*. *vivax*-exposed individuals living in Colombia’s Chocó (Pacific region) and Córdoba (Caribbean region) departments where *P*. *vivax* malaria is endemic [34]. Colombia has areas having unstable malaria transmission levels, an endemic-epidemic transmission pattern and is mainly rural [35]. The selected *P*. *vivax*-exposed individuals were over 18 years-old, had had at least one episode of *P*. *vivax* malaria (the last malarial episode having occurred 6 months previously) and had received treatment. It is known that an *in vitro* cellular response to malarial antigens is suppressed during acute infection; PB samples were taken from exposed individuals [36–38] and thick blood smears were used for confirming that the samples were negative for *P*. *vivax* infection. This study was carried out in line with Colombian Ministry of Health Resolution 8430/1993 (the legal framework for research in Colombia). The individuals were regarded as being at low risk and all data was kept confidentially and rigorously protected. Written informed consent was signed by all individuals prior to sampling; all procedures were evaluated and approved by FIDIC’s ethics committee.

### Study population

This study involved typing 79 *P*. *vivax*-exposed individuals from the aforementioned endemic areas for the HLA-DRβ1 allele; PB samples from 50 individuals living in Bogotá (a non-endemic malaria area) who have never been exposed to malaria were also taken for typing to form the control group. PB samples were taken from *P*. *vivax*-exposed individuals and the control group and placed in EDTA tubes (BD Vacutainer Oakville, ON, CAN). A Wizard Genomic DNA Purification Kit (Promega Corporation, Madison, WI, USA) was used for extracting genomic DNA (gDNA) from 300 μL whole blood, according to the manufacturer’s instructions. The gDNA was verified by 1% agarose gel with SYBR Safe DNA Gel Stain (Thermo Fisher Scientific, CA, USA) and sent to Histogenetics (Ossining, NY, USA) for high-resolution HLA sequence-based typing (SBT) of the β1 locus.

### Experimental groups

HLA-DRβ1 typing was used for determining experimental and control groups. HLA-DRβ1*04, *07, *11 and *13 allele lineages have been reported as having high frequency in malaria endemic areas worldwide [39]; individuals having at least one allele from the allele lineages were thus selected whilst individuals having two of these lineages at the same time were discarded.

### *In silico* selection and chemical synthesis of *Pv*12 B- and T-cell epitopes

Bioinformatics tools were used for selecting B- and T-cell epitopes from the *Pv*12 aa sequence (PlasmoDB PVX_113775 access number). BepiPred 1.0 [19] and ANTHEPROT 2000 v6.0 [40] were used for selecting B-cell epitopes. The NetMHCIIpan-3.1 tool [41] was used for predicting T-cell epitopes, selecting epitopes according to their HLA-DRβ1*0401, *0701, *1101 and *1302 binding profile. IEDB (Immune Epitope Database) [42–44] and TEPITOPE [45] software was used for confirming T-epitopes prediction.

Selected *in silico* peptides and biotinylated control peptides HA (Haemagglutinin Antigen) and TT (Tetanus Toxoid) were synthesised and purified by 21^st^ Century Biochemicals Inc. (Marlborough, MA, USA).

### Immunoblot and immunofluorescence assays (IFA)

PB samples from all endemic area individuals and the control group’s antibody response assays were placed in 6 mL BD vacutainer serum collection tubes (BD Vacutainer). *P*. *vivax* antigens were recognised by separating 50 μg *Pv*12 recombinant protein (r*Pv*12 donated by Moreno-Perez *et al*.) [31] and r*Pv*GAMA (GPI anchored micronemal antigen) control protein (r*Pv*GAMA-CT donated by Baquero *et al*.) [46] on 12% polyacrylamide gel in denaturing conditions; the gel was transferred to a nitrocellulose membrane (Bio-Rad). The membrane was then cut into thin strips and incubated with sera samples diluted 1:50 in blocking buffer. The strips were washed and incubated with a secondary antibody peroxidase anti-human IgG (Vector Labs) at 1:4,000 dilution; a VECTOR VIP peroxidase (HRP) substrate kit (Vector Labs) was used for revealing the assay.The immunofluorescence assays were performed as described by Moreno-Perez *et al*., [31] with some modifications. Briefly, blood samples from individuals who had active *P*. *vivax* infection were spun, the buffy coat removed and the plasma recovered.

*P*. *vivax*-exposed individuals and control group serum samples were diluted 1:50 in TBS-BSA and incubated for 1 hour in a humid chamber. Reactivity was observed by fluorescence microscopy using FITC-labelled (Fluorescein isothiocyanate) anti-human IgG (Sigma-Aldrich) at 1:50 dilution in TBS-BSA. Parasite nuclei were stained with 0.25 μg/mL DAPI (4',6-diamidino-2-phenylindole, dilactate) (Life Technologies) and the slides were then washed with 0.05% TBS-T. The slides were visualised on an Olympus BX51 fluorescence microscope (Olympus Corporation); the images were processed using DP2-BSW software (v2.2, Olympus Corporation) and merged using ImageJ 1.51n software (National Institutes of Health, USA).

### ELISA assays for B-cell epitopes and recombinant proteins

NUNC immuno-modules (Thermo Scientific) were coated with 1 μg of each epitope and each recombinant protein (r*Pv*12 and r*Pv*GAMA) in PBS pH 7.2 and incubated overnight (ON) at 4°C. The sera were incubated in 1:100 dilution in blocking solution for 2 hours and peroxidase anti-human IgG (Vector Labs) was used as secondary antibody in 1:10,000 dilution in blocking solution for 1 hour at RT; 100 μL TMB (3,3′,5,5′-tetramethylbenzidine) peroxidase substrate (Sera-Care) was used for revealing the assay. The reaction was stopped by adding an equal volume of 1M phosphoric acid (H_3_PO_4_) (Merck) and read at 450 nm using a Multiskan GO (Thermo Scientific, Waltham, MA, USA) ELISA reader. The mean for the control group plus two standard deviations was used as cut-off point.

Positive sera were evaluated for IgG subclasses using 3% BSA in PBS-T as blocking solution, following the ELISA protocol described above. Sera were incubated at 1:100 dilution in PBS-T. The following secondary antibodies diluted in PBS-T were used: anti-human IgG1-biotin (clone 8c/6-39) in 1:1,000 dilution, anti-human IgG2-biotin (clone HP-6014) in 1:15,000 dilution, anti-human IgG3-biotin (clone HP-6050) in 1:40,000 dilution and anti-human IgG4-biotin (clone HP-6025) in 1:60,000 dilution (all from Sigma-Aldrich). Pierce high-sensitivity HRP-conjugated (Horseradish peroxidase) streptavidin (Thermo Scientific) in 1:5,000 dilution in PBS, pH 6.8, and TMB (Sera-Care) were used as substrate. The reaction was stopped and read at 450 nm using an ELISA reader.

### Molecular modelling

*Pv12* protein (PlasmoDB PVX_113775 access number) 3D structure was inferred using *Pf12* protein structure (PDB: 2YMO) as template, using the I-TASSER server [47]. The most energetically stable model was selected and the error associated with inferred structure was corrected via molecular dynamics simulations to avoid incorrect atomic contacts. NAMD 2.12 [48] was used for molecular dynamics calculations and CHARMM27 force field for molecular dynamics simulations and analysis [49,50] using periodic boundary conditions. Hydrogen atoms were added using Visual Molecular Dynamics (VMD) *psfgen* plugin [51]. The protein was minimised for 1,000 steps, followed by equilibration for 1 ns; the molecular dynamic simulation was run for 20 ns. Molprobity [52] was used for validating the refined model’s structural quality. VMD was used to locate the B-epitopes studied here in the resulting *pv12* model. The POLYVIEW protein structure visualisation server [53] was used for calculating relative solvent accessibility and secondary structure annotation.

### Purifying anti-HLA-DR monoclonal antibody and HLA-DR complexes

The anti-HLADR monoclonal antibody was obtained from L-243 cell line (ATCC HB-55) culture supernatant [54,55] which was saturated with 45% ammonium sulphate ((NH_4_)_2_ SO_4_) (Merck) for immunoglobulin precipitation and purified by affinity chromatography using Protein A Sepharose.

Four homozygous lymphoblastoid B-cell lines (IHWG) were cultured for each molecule of interest (IHW09025 for HLA-DRβ1*0401, IHW09051 for HLA-DRβ1*0701, IHW09043 for HLA-DRβ1*1101 and IHW09055 for HLA-DRβ1*1302) to obtain HLA-DR complexes. The cells were lysed and molecules purified by affinity chromatography, as described by Vargas *et al*. [56].

Anti-HLADR antibody and HLA-DR purity was verified by 8% polyacrylamide gel and western blot. Amicon Ultra centrifugal filters (Merck) were used for concentrating the positive fractions and quantified using a Micro BCA Protein Assay Kit (Thermo Scientific) which were then stored at -80°C until use.

### *In vitro* HLA-DRβ1 binding assays and IC50 values

Selected *in silico* peptides’ HLA-DRβ1 binding affinity was evaluated by measuring biotinylated control peptide displacement. Biotinylated peptide HA_306-318_ (PKYVKQNTLKLAT) [23,57] was used as control peptide for assays with HLA-DRβ1*04 and *11 whilst biotinylated peptide TT (QYIKANSKFIGITE) [58] was used for assays with HLA-DRβ1*07 and *13.

The selected peptides’ *in vitro* binding capacity was evaluated by competitive ELISA, as described by Vargas *et al*., [56] and Saravia *et al*. [23]. Briefly, a complex involving 0.1 μM purified molecules, 5 μM biotinylated control peptide and 250 μM of the peptide to be evaluated (50-fold excess of control peptide) was incubated for 24 hours at RT. The complex was then placed on NUNC-Immuno MicroWell (Thermo Scientific) plates pre-coated with 10 μg/mL anti-HLA-DR, incubated ON at 4°C and blocked with 3% PBS-BSA for 2 hours at RT. The wells were incubated with alkaline phosphatase streptavidin reagents (Vector Labs) in 1:500 dilution in PBS and subsequently revealed with 200 μL p-NPP (p-nitrophenylphosphate) substrate (Sigma-Aldrich) and read at 405 nm on a MultiSkan GO (Thermo Fisher Scientific, Waltham, MA, USA) ELISA reader. Binding percentages were calculated by using the following formula: 100*[1-(OD in the presence of competitor / OD in the absence of competitor)].

IC50 values were calculated for peptides having more than 50% binding; the aforementioned protocol was followed, varying the concentrations of the peptides to be evaluated by using 4.2 to 250 μM serial dilutions. Binding values were calculated by using the following formula: 1-(OD in the presence of competitor / OD in the absence of competitor). The resulting values were plotted using a second order exponential decay curve using a Mathematica (Wolfram Research, Inc., Mathematica, version 11.0, Champaign, IL (2016)) software formula to calculate the concentration at which the peptide to be evaluated displaced biotinylated control peptide by 50%.

### Lymphoproliferation and cytokine quantification assays

*P*. *vivax*-exposed individuals and control group PB samples were taken and placed in citrate phosphate dextrose (CPD) solution in Vacutainer tubes (BD Vacutainer); 40 mL blood was used to obtain PBMCs from gradient centrifugation with Ficoll-Paque PLUS (GE Healthcare) to evaluate the proliferative response against *Pv*12 peptides.

Two hundred thousand (2 x 10^5^) PBMCs per well were seeded in 96-well round-bottomed plates (Costar, Corning Incorporated) in RPMI 1640 medium (Gibco) supplemented with inactivated 10% autologous plasma, 2 g/L sodium bicarbonate, 2 mM L-glutamine, 1 mM sodium pyruvate and100 U/mL Antibiotic-Antimycotic solution (all Gibco). The PBMCs were labelled with carboxyfluorescein diacetate N-succinimidyl ester (CFSE) (5μM) (CellTrace CFSE cell proliferation kit, Molecular Probes, Eugene, OR, USA) and stimulated with each peptide at 10 μg/mL final concentration.

Control PBMCs were stimulated with 5 μg/mL parasite lysate and 2% phytohaemagglutinin (PHA) (Sigma) as positive controls. Low binding peptide (LBP) 39115 was used as negative control in experimental binding assays and a well containing PBMCs with just medium (unstimulated) was used as assay control. The cells were cultured at 37°C in a 5% CO_2_ atmosphere for 96 hours; assays were carried out in duplicate. Pacific Blue-labelled mouse anti-human CD4 (RPA-T4 clone) (BD Biosciences) antibody was then used for labelling the cells which were read on a FACS Canto II flow cytometer (BD Biosciences). FlowJo software v7.6.5 (FlowJo, LLC) was used for analysing the data and CD4^+^ T-lymphocyte proliferation.

Cell proliferation was calculated by relative loss regarding CFSE fluorescence intensity, calculated as the percentage of fluorescence intensity of cells co-cultured with antigen divided by the percentage of co-cultured cells’ fluorescence intensity in the absence of antigen [59]. The results were expressed as stimulation index (SI); a ≥2 value was considered positive.

CD4+ lymphocyte mean fluorescence intensity (MFI) values were used for additional analysis. Cell proliferation was calculated as the stimulation rate, where the unstimulated CD4+ T-cell population MFI was divided by the stimulated CD4+ T-cell population’s MFI. MFI becomes less as the antigen stimulates CD4+ T-lymphocytes, whereby ≥1 values indicate cell proliferation [60]. The relative value of proliferation was considered positive, with at least 1% loss in the mean intensity of florescence of the CD4+ population, i.e. ≥1.01.

The supernatants were collected and stored at -80°C. A Human cytometric bead array (CBA) Th1/Th2 Cytokine Kit II (BD Biosciences) was used for measuring cytokines, following the manufacturer’s instructions. Samples were read on a FACS Canto II flow cytometry and FCAP Array software v3.0.1 (BD Biosciences) was used for analysing cytokine results (expressed in pg/mL) and sample values were compared to those for wells without stimulation. The cut-off point was calculated as control group mean plus two standard deviations.

### Analysing the data

GraphPad Prism v5.01 software (GraphPad Software, Inc.) was used for analysing and plotting the data. The Shapiro-Wilk test was used for tests of normality, the Wilcoxon-Mann-Whitney U-test for comparing two non-parametric variables and Student’s t-test for variables having normal distribution. The Kruskal-Wallis test with Dunn’s post-test was used for multiple comparisons. Spearman’s coefficient (ρ = Rho) was used for calculating correlations between experimental and clinical data. A 95% confidence interval and *p*≤0.05 were set as being significant; significance level has been highlighted by asterisks on all graphs, as follows: (*) *p*<0.05, (**) *p*<0.005 and (***) *p*<0.0005.

## Results

### Study area and study population

**Fig 1:**
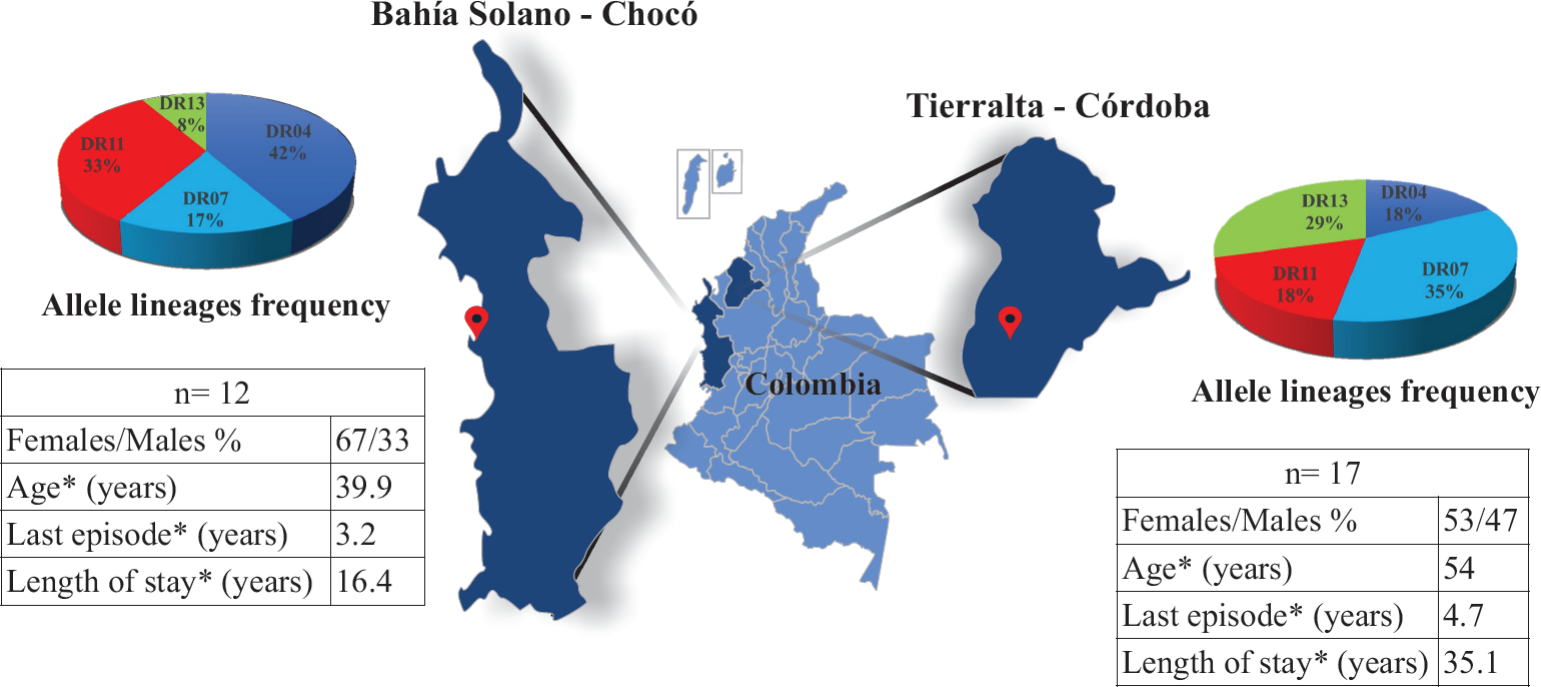
Study area and study population’s demographic parameters. The allele lineage frequency of exposed individuals from endemic areas in Colombia (Chocó and Córdoba departments); n = amount of individuals; *mean value for age, time of last episode and length of stay.

Around 70% of malarial cases in Colombia are caused by *P*. *vivax* [35]; *P*. *vivax*-exposed individuals living in Bahía Solano (Chocó) and Tierralta (Córdoba) were thus selected (Fig 1). People living in Colombia’s Chocó and Córdoba departments have higher *P*. *vivax* infection incidence; this was reported by the Colombian Public Health Surveillance System (Sistema de Salud Pública–SIVIGILA) in its annual report for 2015 to 2017 [34]. Exposed individuals from these departments were thus selected for this study.

Two samples were needed from the study population for evaluating their immune responses; the first sample was used for typing the HLA-DRβ1* gene in the experimental and control groups. Four experimental groups were clustered according to the selected allele linages: HLA-DRβ1*04 (n = 8), HLA-DRβ1*07 (n = 8), HLA DRβ1*11 (n = 7) and HLA-DRβ1*13 (n = 6). The second sample was used for determining T- and B-cell antigenicity, analysing the immune responses of the individuals who had not been infected for at least 6 months to find memory T-cells and long-lived antibodies against conserved peptides.

### *P*. *vivax*-exposed individuals’ antibody reactivity

Western blot was used for evaluating r*Pv*12 and r*Pv*GAMA protein reactivity for determining their antigenicity; r*Pv*12 was recognised by 86% of sera and r*Pv*GAMA (control protein) by 85% of sera from thirty *P*. *vivax*-exposed individuals (S1A Fig). Control group sera samples were evaluated, no reactivity being observed. *P*. *vivax-*exposed individuals and control group samples were used for IFA assays. FITC fluorescence was detected in parasitised red blood cells (pRBC) when reacting with the thirty exposed individuals’ serum; conversely, no reactivity was observed in the control group’s serum samples (S1B Fig).

### Antibody response against recombinant proteins and B-cell epitopes

**Fig 2:**
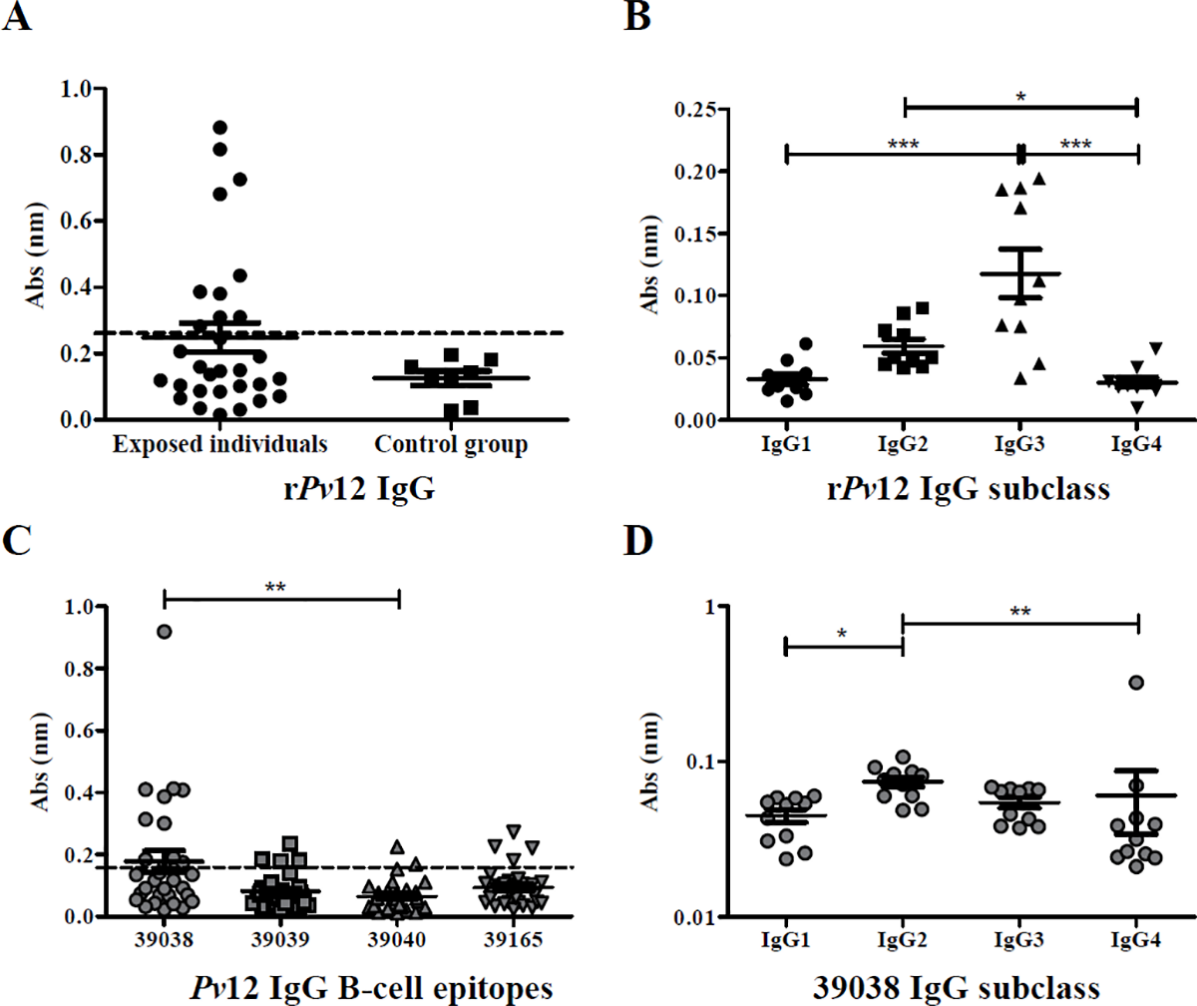
Humoral immune response against *Pv*12. **A. IgG antibody response against r*Pv*12**. Seropositive samples (n = 30) were those above the cut-off point (0.260, dotted line), calculated as control group’s mean plus two standard deviations. **B. Analysing IgG subclass response to r*Pv*12.** The Kruskal-Wallis test was used for analysing differences between each IgG subclass response in *P*. *vivax*-exposed individuals’ samples. **C. IgG antibody response against *Pv*12 B-cell epitopes (n = 30).** Seropositive samples were those above the cut-off point (0.159, dotted line), calculated as control group’s mean plus two standard deviations. **D. Evaluating IgG subclass response to the 39038 epitope (n = 11).** The Kruskal-Wallis test was used for analysing differences between each IgG subclass response in *P*. *vivax*-exposed individuals’ samples. (*) *p*<0.05, (**) *p*<0.005 and (***) *p*<0.0005.

Evaluating the reactivity of natural antibodies from the sera samples taken from the 30 malaria-exposed individuals revealed that 33.3% (10 positive samples) had IgG antibodies against r*Pv*12 (0.2480 mean absorbance); no control group sera recognised r*Pv*12. No significant differences were observed between exposed and control groups (Fig 2A). Regarding IgG subclass, IgG3 significantly predominated over IgG1 and IgG4 antibodies; significant differences were found between IgG2 and IgG4 non-cytophilic antibodies (*p*<0.0001) (Fig 2B). r*Pv*GAMA was used as positive control (0.4757 mean absorbance); 70% (21 samples) were seropositive and significant differences were observed regarding the control group (*p* = 0.0004) (S2A Fig).

Bioinformatics tools were used for selecting four B-cell epitopes for analysing the humoral immune response. IgG antibody response against selected *Pv*12 B-epitopes was evaluated in sera from individuals exposed to natural *P*. *vivax* infection. The highest amount of seropositive samples (11 samples / 36.6%) was related to peptide 39038 followed by 39039 and 39165 (4 samples each / 13.3%), and 39040 (2 samples / 6.6%) (Fig 2C).

Peptide 39038 had the highest mean absorbance (0.1780), significant differences being observed regarding 39040 which had the lowest mean absorbance (0.06581) out of all four peptides. The Kruskal-Wallis test (with Dunn’s multiple comparison post-test) was used for statistical analysis; there were significant differences regarding epitope response (*p* = 0.0027) (Fig 2C). Although there were no statistically significant differences between exposed individuals and control groups, the exposed individuals had a greater response.

ELISA *Pv*12 peptide results were analysed by endemic area, samples from the Chocó department having greater response compared to those from the Córdoba department. Positive sera reacted to the four peptides, 61.5% of such samples were from the Chocó department. The Mann-Whitney test gave a significantly higher response for peptides 39039 (*p* = 0.0028), 39040 (*p* = 0.0045) and 39165 (*p* = 0.0020) in exposed individuals from the Chocó department compared to exposed individuals from the Córdoba department (S2B Fig).

Seropositive samples were selected for evaluating IgG subclasses; significant differences were only observed for peptide 39038 between IgG subclasses (*p* = 0.0012) whilst other peptides had no significant differences. 39038 had a clear IgG2 predominance regarding other subclasses, having statistically significant differences with IgG1 and IgG4 (Fig 2D).

Using Spearman’s test for analysing correlation for non-parametric data revealed tendencies associated with antibody response. One parameter evaluated was time elapsed (in years) since the last episode where decreased antibody response to epitopes 39039 (Rho = -0.40, *p* = 0.044), 39040 (Rho = -0.44, *p* = 0.024) and 39165 (Rho = -0.49, *p* = 0.010) was observed as time elapsed. Reduced response was also observed for *Pv*GAMA protein control (Rho = -0.41, *p* = 0.038).

The other parameter evaluated was exposed people’s age, a tendency for decreased antibody response to epitopes 39039 (Rho = -0.44, *p* = 0.025) and 39165 (Rho = -0.44, *p* = 0.025) being observed as age increased.

### *Pv*12 T-cell epitope i*n silico* selection

**Fig 3:**
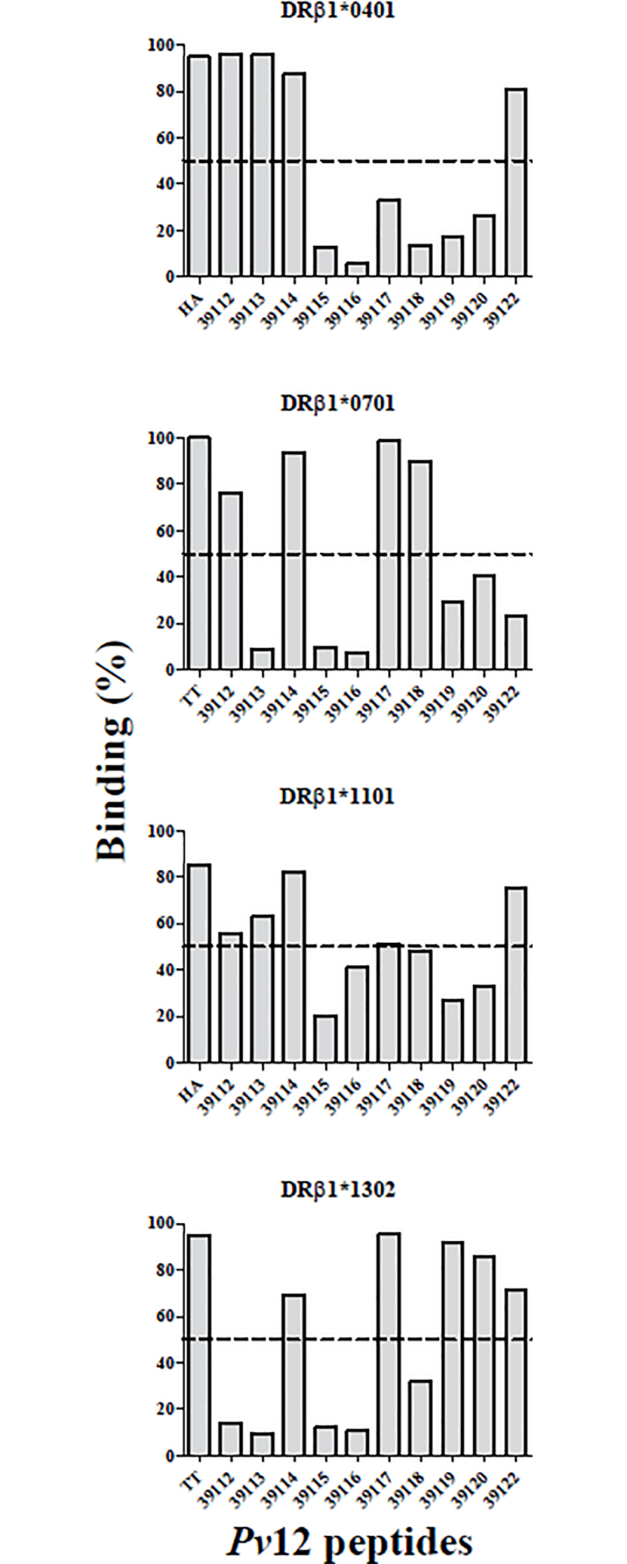
*Pv*12 peptide *in vitro* binding to purified HLA-DRβ1* molecules. A cut-off line is shown at 50% binding for selecting HBP for further evaluation of IC50 value. Each plot shows percentage epitope HLA-DRβ1* binding in this study and that for their control peptides.

NetMHCIIpan-3.1 was used for selecting ten epitopes from the *Pv*12 aa sequence for HLA-DRβ1*0401, *0701, *1101 and *1302 alleles. These peptides were then evaluated in *in vitro* binding assays, using the same alleles in this study; those having >50% binding were chosen as high-binding peptides (HBP). *In vitro* results showed that 4/10 peptides bound to the HLA-DRβ1*0401 allele (46.94% mean binding), 4/10 peptides bound to HLA-DRβ1*0701 (47.81% mean binding), 5/10 peptides bound to HLA-DRβ1*1101 (49.54% mean binding) and 5/10 peptides bound to HLA-DRβ1*1302 (49.17% mean binding) (Fig 3).

**Fig 4:**
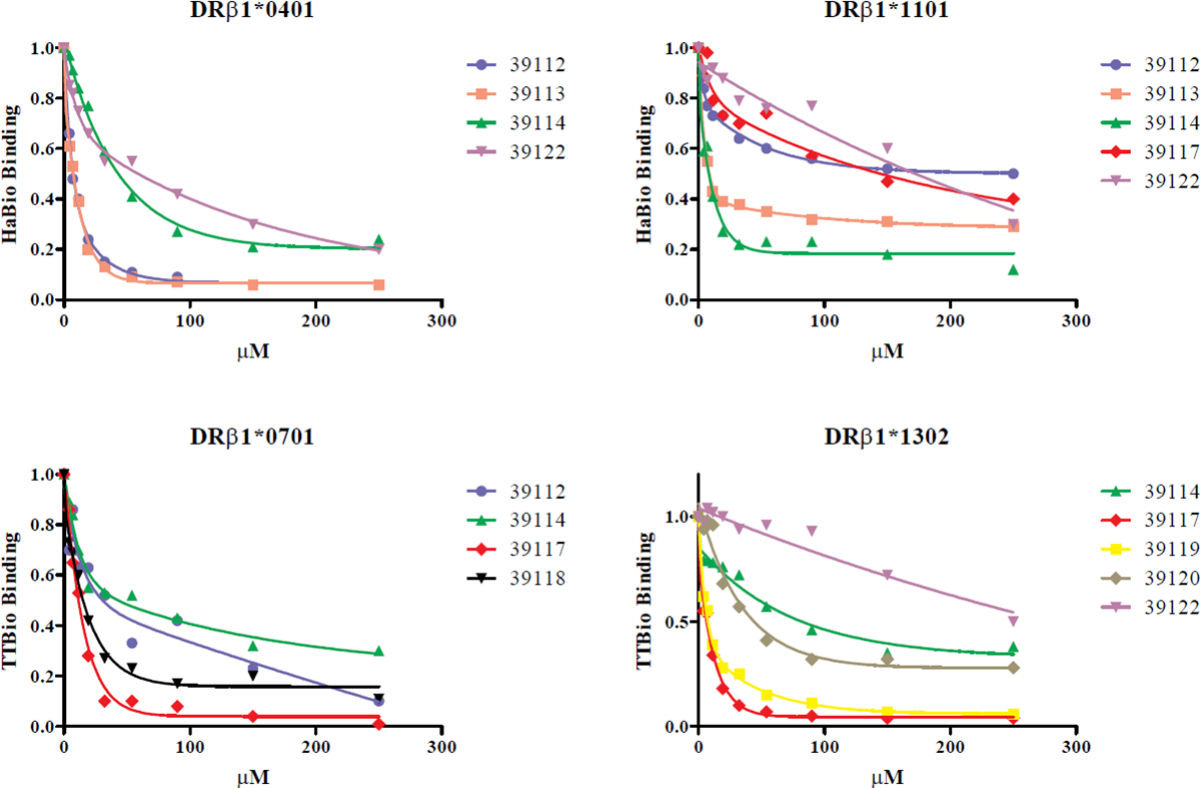
*In vitro* assays for calculating a *Pv*12 peptide’s IC50 value. Different epitope concentrations were evaluated for calculating the value at which control peptide was displaced by 50% (using a second order exponential decay function). Each point under the curve represents evaluated epitope concentration-dependent control peptide binding (μM).

The IC50 ratio values were calculated from the IC50 value (μM) for each peptide (Fig 4) divided by the IC50 value (μM) of its control peptide; ≤10 values were considered good binding peptides (GBP). Experimental assay values agreed 77.5% with the *in silico* prediction for our alleles (Table 1). The peptides having the lowest IC50 values (μM) were selected for the lymphoproliferative assays, peptide 39113 for HLA-DRβ1*04 and *11 allele linages and peptide 39117 for HLA-DRβ1*07 and *13 allele linages. Peptide 39114 was considered a universal peptide (UP) because it was the only one displaying ≥50% binding to the four alleles evaluated here.

**Table 1:** T-cell epitopes selected *in silico* and *Pv*12 *in vitro* binding.

Peptide code	Sequence	CoreF	HLA-DRβ1* allele	NetMHCIIpan- 3.1 (%rank)^a^	Binding percentage^b^	IC50 μM^b^	IC50 ratio
39112	RKNYELLPNNCFEQV	YELLPNNCF	DRβ1*0401	3.5	95.78	7.26	0.37
			DRβ1*0701	14.0	76.44	29.23	1.25
			DRβ1*1101	35.0	55.44	250	52.41
			DRβ1*1302	30.0	14.09	ND	ND
39113	EECFLQGFNLSGKKE	FLQGFNLSG	DRβ1*0401	3.0	95.81	7.36	0.38
			DRβ1*0701	24.0	8.89	ND	ND
			DRβ1*1101	27.0	62.88	38.54	8.08
			DRβ1*1302	55.0	9.28	ND	ND
39114	YNKIFYARVPQRIYQ	IFYARVPQR	DRβ1*0401	8.5	87.38	43.18	2.22
		FYARVPQRI	DRβ1*0701	0.7	93.87	43.09	1.84
		IFYARVPQR	DRβ1*1101	0.9	82.09	8.28	1.74
		FYARVPQRI	DRβ1*1302	8.5	69.37	73.30	9.83
39115	KKSYDDVSFRVPPNL	SYDDVSFRV	DRβ1*0401	65.0	12.98	ND	ND
			DRβ1*0701	65.0	9.86	ND	ND
		DVSFRVPPN	DRβ1*1101	75.0	20.02	ND	ND
		SYDDVSFRV	DRβ1*1302	60.0	12.00	ND	ND
39116	NKAKIRVRKRSGEEY	IRVRKRSGE	DRβ1*0401	95.0	5.76	ND	ND
		KAKIRVRKR	DRβ1*0701	85.0	7.33	ND	ND
			DRβ1*1101	13.0	41.12	ND	ND
		IRVRKRSGE	DRβ1*1302	85.0	10.50	ND	ND
39117	LGIIEVLIPSLPKKI	IEVLIPSLP	DRβ1*0401	14.0	33.08	ND	ND
		LIPSLPKKI	DRβ1*0701	8.0	98.56	11.51	0.49
		EVLIPSLPK	DRβ1*1101	9.5	51.13	133.77	28.04
		LIPSLPKKI	DRβ1*1302	11.0	95.42	6.65	0.89
39118	LVEYLHGAAAIVKRK	YLHGAAAIV	DRβ1*0401	3.0	13.48	ND	ND
			DRβ1*0701	1.2	90.02	15.18	0.65
		LHGAAAIVK	DRβ1*1101	11.0	48.21	ND	ND
		YLHGAAAIV	DRβ1*1302	7.0	31.71	ND	ND
39119	GCDFTKNTSPLFTKG	FTKNTSPLF	DRβ1*0401	6.5	17.45	ND	ND
			DRβ1*0701	7.5	29.45	ND	ND
			DRβ1*1101	30.0	26.94	ND	ND
			DRβ1*1302	4.5	92.18	7.47	1.00
39120	LTDLVMDHYNKIFYA	LVMDHYNKI	DRβ1*0401	31.0	26.73	ND	ND
			DRβ1*0701	37.0	40.62	ND	ND
			DRβ1*1101	36.0	32.68	ND	ND
			DRβ1*1302	3.5	85.99	38	5.09
39122	KRLVAHFEFATTPDD	FEFATTPDD	DRβ1*0401	40.0	80.94	58.89	3.03
		LVAHFEFAT	DRβ1*0701	65.0	23.08	ND	ND
		VAHFEFATT	DRβ1*1101	65.0	74.87	170	35.64
		LVAHFEFAT	DRβ1*1302	85.0	71.13	250	33.51


Peptide codes and sequences are shown, as well as NetMHCIIpan-3.1 predicted HLA-DRβ alleles’ core and % rank. IC50 was assessed for peptides having ≥50% binding; IC50 values were expressed in μM and peptides were considered GBPs when IC50 ratio was ≤10 (IC50 peptide/IC50 control peptide).

^**a**^%rank values were considered as follows: LBP≥2 to ≤10 and GBP ≤2. ND (not determined) means that a peptide had less than 50% binding, so that its IC50 value was not evaluated.

^**b**^Data from this study; IC50 values were calculated for each control peptide with each DRβ1* allele. Control HA-DRβ1*0401 IC50 = 19.44 μM, TT-DRβ1*0701 IC50 = 23.37 μM, HA-DRβ1*1101 IC50 = 4.77 μM, TT-DRβ1*1302 IC50 = 7.46 μM. Peptides having ≤10 IC50 ratio were considered GBP.

### Experimental groups’ lymphocyte proliferation

Exposed individuals and control groups’ CD4^+^ T-cell proliferative responses stimulated with UP 39114, GBP 39113 and 39117 were evaluated; LBP 39115 was used as negative control and *P*. *vivax* lysate and PHA as positive controls.

**Fig 5:**
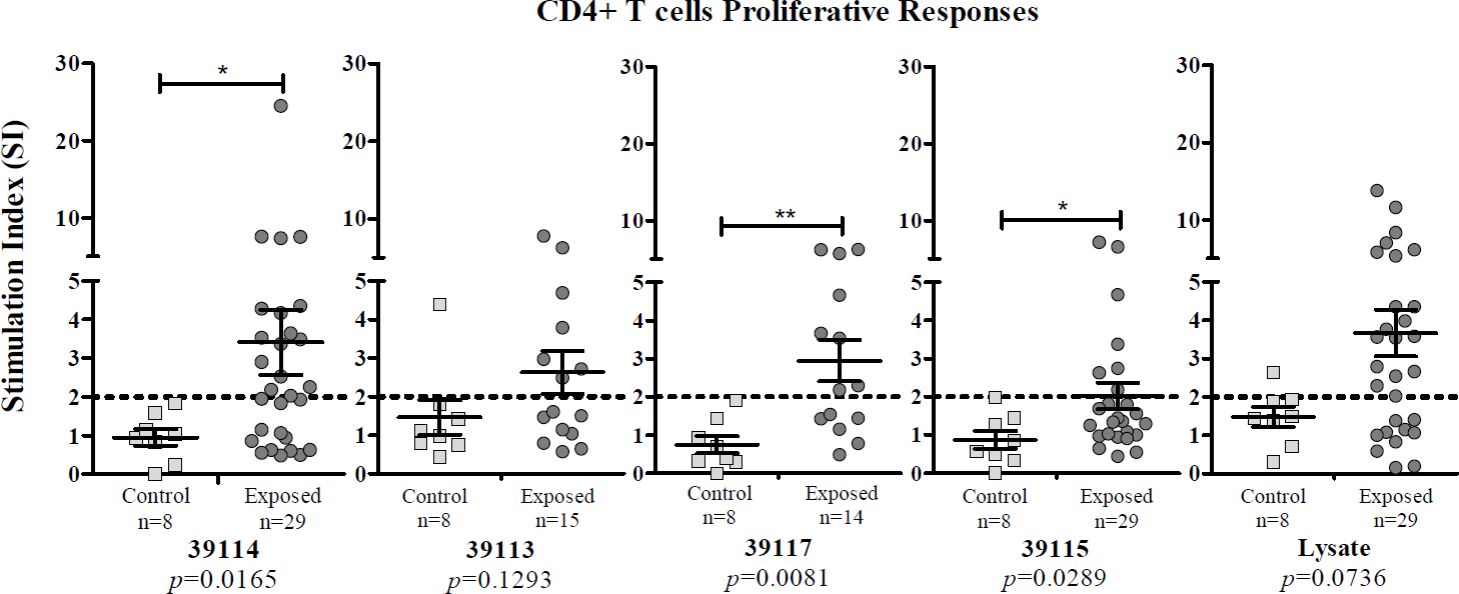
Lymphocyte proliferative response to *Pv*12 epitopes from individuals exposed to *P*. *vivax* infection compared to control group. UP 39114 (n = 29); DRβ1*04 and DRβ1*11 GBP 39113 (n = 15); DRβ1*07 and DRβ1*13 GBP 39117 (n = 14); LBP / control 39115 (n = 29) and *P*. *vivax* lysate (n = 29) responses are shown. Mann-Whitney and Student’s t-tests were used for assessing statistically significant differences between exposed individuals and control group responses. (*) *p*<0.05 and (**) *p*<0.005.

Exposed individuals having ≥2 stimulation index (SI) were considered responders. Average response to UP 39114 was 3.409 (SE = 0.8458), 2.632 (SE = 0.5563) to GBP 39113, 2.947 (SE = 0.5449) to GBP 39117, 2.018 (SE = 0.3478) to LBP 39115 and 3.664 (SE = 0.6072) to lysate. There was no proliferation in the control group when cells were stimulated with the peptides or *P*. *vivax* lysate (according to the SI observed) (Fig 5).

Exposed individuals’ frequency of response to UP 39114 was 55.2%, 46.7% to GBP 39113, 57.1% to GBP 39117, 28% to LBP 39115 and 65.5% to lysate. Control group response to UP 39114 and GBP 39117 was 0/8, 1/8 to GBP 39113, 0/8 to LBP 39115 and 1/8 to lysate.

There were statistically significantly differences between exposed individuals and control groups regarding UP 39114 (*p* = 0.0165), GBP 39117 (*p* = 0.0081) and LBP 39115 (*p* = 0.0289).

*P*. *vivax* lysate induced a greater proliferative response in the exposed individuals (mean SI = 12.47 ± 1.636 SE); however, no statistically significant differences were observed when compared to the control group (mean SI = 5.45 ± 1.138 SE) (Fig 5). All PHA-stimulated PBMCs from exposed individuals and the control group had positive SI, having statistically significant differences (*p* = 0.0103). It was found that 96.6% of the exposed individuals and 100% of the control group responded to PHA (S3 Fig).

Analysing relative proliferation values calculated from MFI data gave similar results, thereby validating data obtained using FlowJo software. There was a maximum 12% variation regarding the amount of exposed responders for lysate, statistical differences being maintained between the exposed and control groups regarding peptides 39114 and 39117. The greatest difference between both analyses was found for LBP 39115 stimulation which gave 50% responders according to relative proliferation values; however, it had a low mean score/value regarding other antigens and had no statistical differences regarding the control group. No mean relative value for the control group exceeded the cut-off line after stimulation with the different antigens, thereby agreeing with FlowJo software results.

### Experimental groups’ cytokine profiles

**Fig 6:**
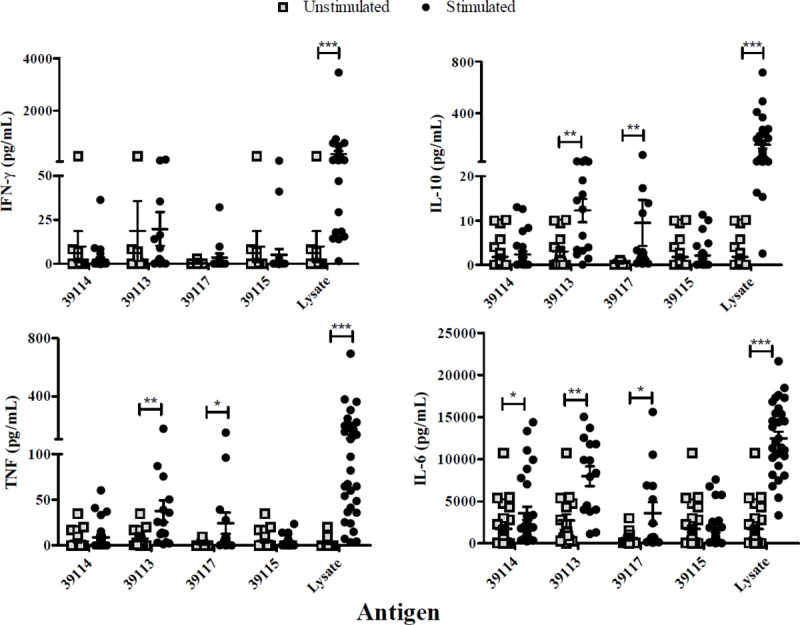
Exposed individuals’ supernatant culture *in vitro* cytokine production. Individual data shows the mean value of non-stimulated and PBMCs stimulated with UP 39114, GBP 39113 and 39117 and *P*. *vivax* lysate. IFN-γ, TNF, IL-10 and IL-6 levels were measured by CBA kit; cytokine concentration is expressed in pg/mL. Statistically significant differences (*p*≤0.05) are shown and data represents the mean ± SEM (standard error media) for all values. (*) *p*<0.05, (**) *p*<0.005 and (***) *p*<0.0005.

Statistical analysis regarding exposed individuals’ unstimulated and stimulated PBMCs showed that IFN-γ (Interferon gamma) production was only significant after stimulation with *P*. *vivax*-lysate (*p* = 0.0001). TNF (Tumor Necrosis Factor) production was significantly different for GBP 39113 (*p* = 0.0048), GBP 39117 (*p* = 0.0183) and *P*. *vivax*-lysate (*p* = 0.0001). IL-10 (Interleukin 10) had higher production with GBP 39113 (*p* = 0.0054), GBP 39117 (*p* = 0.0012) and *P*. *vivax*-lysate (*p* = 0.0001). IL-6 (Interleukin 6) responses were significantly greater to UP 39114 (*p* = 0.0108), GBP 39113 (*p* = 0.0025), GBP 39117 (*p* = 0.0366) and *P*. *vivax*-lysate (*p* = 0.0001) (Fig 6).

**Fig 7:**
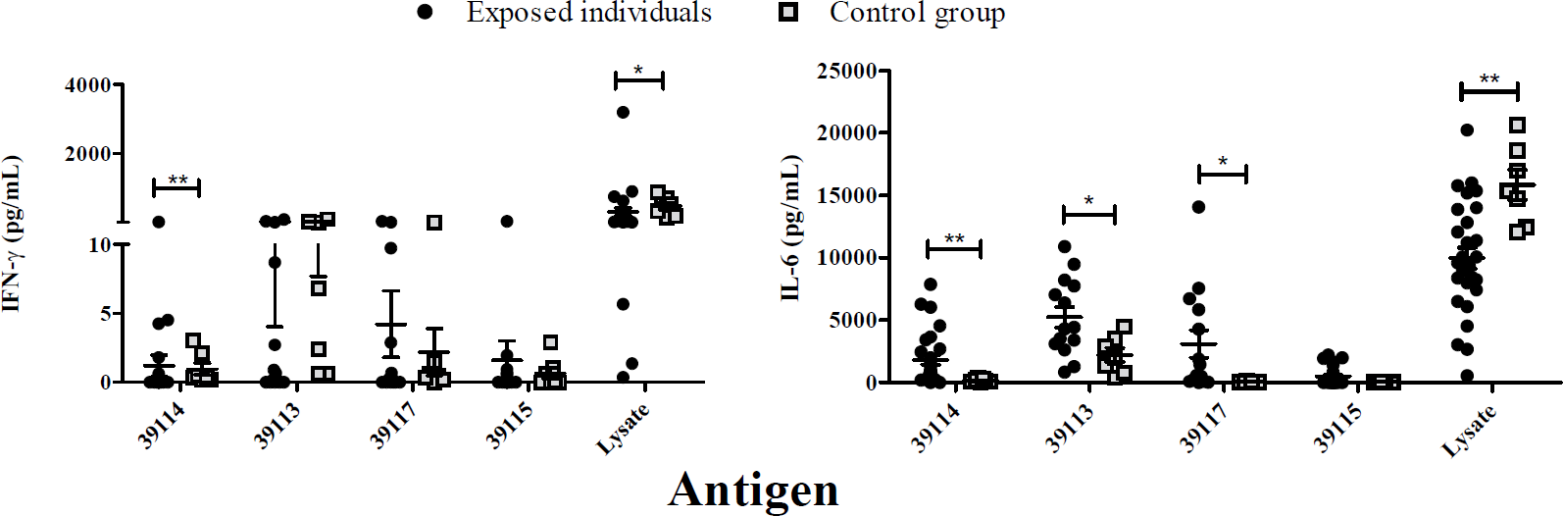
Exposed individuals and control group supernatant culture *in vitro* cytokine production. Individual cytokine values from PBMCs stimulated with UP 39114, GBP 39113 and 39117 and *P*. *vivax* lysate. IFN-γ and IL-6 levels were measured by CBA kit and cytokine concentration is expressed in pg/mL. Statistically significant differences (*p*≤0.05) are shown and data is the mean ± SEM for all values. (*) *p*<0.05 and (**) *p*<0.005.

*P*. *vivax*-exposed individuals and control groups’ cytokine levels were compared, significant differences being found regarding IFN-γ production for UP 39114 (*p* = 0.0036) and *P*. *vivax* lysate (*p* = 0.0246). Significant differences regarding IL-6 production were found for UP 39114 (*p* = 0.0021), 39113 (*p* = 0.0253), 39117 (*p* = 0.0101) and *P*. *vivax* lysate (*p* = 0.0028) (Fig 7).

### *Pv*12 structure modelling

**Fig 8:**
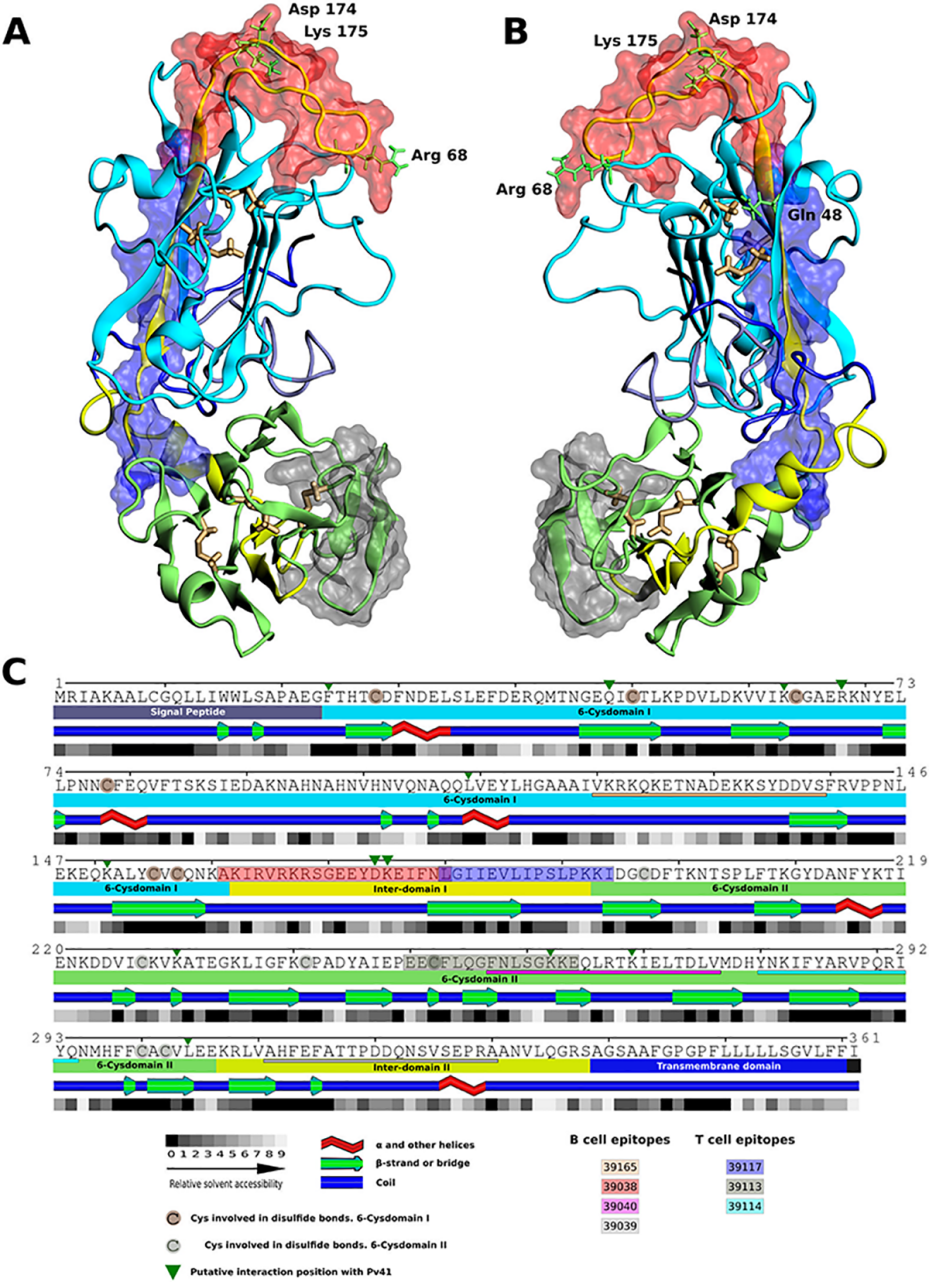
*Pv*12 structure model. A (top view) and B (bottom view). *Pv*12 3D homology model. B-cell epitope 39038 (red) and T-cell epitopes 39117 (blue) and 39113 (grey) are shown as transparent surfaces. Potential interacting positions with *Pv*41 are shown in green and conserved Cys residues in yellow. C. *Pv*12 regions and primary/secondary structure representation, including relative solvent accessibility and potential interacting residues with *Pv*41 (green triangles). Best epitopes are shown (39038- red box, 39117-blue box, 39113-gray box). Other B- and T-cell epitopes are underlined. Conserved cysteine residues are shaded in brown.

*Pv*12 structure was modelled using a homology approach using the *Pf*12 structure model as template. Overall the identity between both proteins was 37% with a similarity of 56% (S1 File). The best I-TASSER model was refined using molecular dynamics, and the best B- and T-cell epitopes (39038, 39117 and 39113) were mapped over the 3D model (Fig 8A and 8B). The remaining epitopes were mapped on the primary sequence and secondary structure model (Fig 8C). All B-cell epitopes were found in high solvent accessibility regions of the protein, in contrast, T-cell epitopes were found in low solvent accessibility regions. Two favourable regions were found combining B- and T-cell epitopes, one constituted by the B-cell epitope 39038 and the T-cell epitope 39117 (Inter-domain I, residues 161 to 194, Fig 8) and the other comprises the B-cell epitope 39040 and the T-cell epitope 39113 (6-Cys domain II, residues 250 to 276, Fig 8). In the first region, both the B-cell and the T-cell epitopes were involved in good responses, however, in the second region, only the T-cell epitope was suitable, whilst the B-cell epitope showed a low response.

Potential interaction positions between *Pv*12 and *Pv*41 were also mapped (Fig 8C, green triangles) based on the binding regions previously determined between *Pf*12 and *Pf*41 [61,62]. *Pf*41*/Pv*41 were also orthologous proteins (as *Pf*12*/Pv*12), showing an identity of 43% and a similarity of 60% (S1B File). *Pf*12*/Pf*41 interactions involved charged residues, and two were mapped directly in the 39038–39117 region (Asp 174 and Lys 175 into 39038 B-cell epitope) and other two less than 3Å from this region (Gln 48 and Arg 68) (Fig 8). Two other potential interaction positions were mapped into the 39113–39040 region. These could indicate a potential blocking activity of antibodies generated against 39038–39117 *Pv*12 region and 39113–39040 region.

## Discussion

A particular characteristic of the six-cysteine (Cys6) protein family is that it is expressed during all parasite life-cycle stages (mature Mrz, gametocytes and pre-erythrocyte stages), making such proteins interesting vaccine candidates [63]. The *Pv*12 surface protein is a member of this family, expressed in blood stage and has a GPI anchor; it is one of the most conserved antigens amongst *Plasmodium* species and *P*. *vivax* isolates [33]. *Pv*12 is homologue of the *Pf*12 protein in *P*. *falciparum* and (together with other proteins) has been suggested as a possible ligand during Mrz invasion of target cells [64]; it is also recognised by the serum from patients exposed to natural infection [32]. People from/living in *P*. *falciparum* malaria-exposed endemic areas develop antibody responses to GPI proteins, correlating with protection against the disease’s symptoms, such as fever and anaemia [65].

r*Pv*12 immunoblot assays led to ascertaining *Pv*12 recognition by sera from individuals naturally exposed to *P*. *vivax* infection, indicating that is capable of triggering a memory-mediated humoral immune response following infection. These results confirmed this protein’s antigenic role and agreed with the studies carried out by Li *et al*., and Moreno *et al*. [30,31].

Evaluating ELISA recognition of the whole *Pv*12 in this study showed 33% positivity; likewise, other studies carried out by Chen *et al*. and Li *et al*., involving the protein array technique, found that 15% and 49% of exposed people’s sera recognised *Pv*12, respectively [9,30].

Although exposed individuals’ sera reactivity to r*Pv*12 and to B-cell epitopes was relatively low, a clear tendency was observed involving greater reactivity by exposed sera, unlike that of the control group. This agreed with a study by Jangpatarapongsa *et al*., who reported low anti-*P*. *vivax* antibody levels but also highlighted immune individuals having specific reactivity against the parasite [66].

Exposed individuals’ sera average IgG response to r*Pv*12 was greater than the mean response to the B-epitopes evaluated here. Differences were observed regarding IgG subtypes when evaluating IgG subclasses’ response to the recombinant protein and B-epitopes. A clear tendency regarding IgG3 response to total protein was observed whilst there was high IgG2 response to B-epitopes regarding the low response to IgG4. This type of response (IgG2/IgG4) has been reported previously and has been associated with a protection and resolution role concerning *P*. *falciparum* infection and protection in that caused by *P*. *vivax* [67,68].

Epitope 39038 had the best recognition of naturally-acquired antibodies of the four B-epitopes evaluated in this study; previous studies have reported it as being as immunogenic in rabbits [31]. This peptide can be considered a good humoral immune response inducer and is proposed as a vaccine candidate due to its potentially protective role.

Lymphoproliferation assay results showed that the *Pv*12-derived peptides selected for their HLA-DR binding were capable of inducing/triggering a lymphoproliferative response when recognised by memory T-cells. Such response specificity was confirmed when comparing such results to those for the control group; although they had the same allele lineages as the exposed group, they did not have a proliferative response.

Other studies have recorded high stimulation values when evaluating the proliferative response of complete proteins or allergens in constant contact with the immune system [60,69]. Although such values were low in this study, the results were similar to those reported by Martinez *el at*., who used ~20 aa length peptides as antigenic epitopes [22]; this highlights the evaluated peptides’ importance which (despite accounting for just a minimal fraction of *Pv*12) were capable of triggering a memory T-cell response.

UP 39114 and GBP 39117 were selected according to *in vitro* binding assays, as well as having a ≥55% proliferative response (with significant differences regarding the control group) and a mean SI≥2. These results indicated a specific T-cell response for *Pv*12 epitopes and ascertained these epitopes’ antigenicity when recognised by immune system memory cells. They correlated proliferation and cytokine production, as seen in other studies with *Plasmodium* antigens [37,70,71].

The PBMCs stimulated with GBP 39113 and 39117 triggered IL-6, IL-10 and TNF production. IL-6 production has been associated with cell proliferation and differentiation [72], agreeing with our results where peptides (two GBP, the UP and parasite lysate) had a significant increase in this cytokine. This cytokine is considered multifunctional regarding an immune response. IL-6, IL-10 and TNF production has been associated with thrombocytopenia in *P*. *vivax* malaria patients [73]. IFN-γ production is associated with protection against pre-erythrocyte malarial stages through both *in vitro* and *in vivo* nitric oxide (NO) production following infection with *P*. *falciparum*, *P*. *berghei*, *P*. *yoelii* or *P*. *chabaudi* Spz [74–76]. Several studies have highlighted the role of proinflammatory cytokines as mediators of protective immunity against malaria, including TNF which, together with IFN-γ, acts synergistically to optimise NO production which is important for parasite destruction [77]. IFN-γ, together with other pro-inflammatory cytokines, participates in inhibiting parasite development within hepatocytes and macrophage activation in intra-erythrocytic parasite phagocytosis or as free Mrz [78].

IL-10 was another cytokine having significant differential production after being stimulated with GBP 39113 and 39117. Recent studies have shown that memory CD4+ lymphocytes proliferate during secondary malarial infection, producing an immune response which controls parasitic load and rapidly produces IL-10 regulating inflammation. This demonstrates IL-10-producing CD4+ lymphocytes’ importance in secondary infections thereby influencing the nature of immune anamnestic responses during secondary malarial infection [79].

Previous studies have shown that PBMCs from naive individuals stimulated with *P*. *falciparum* parasitised RBC [80] or stimulated with malaria antigens did produce IFN-γ [81], mainly innate cells, such as natural killer cells. PBMCs from non-exposed individuals in another study produced IFN-γ and IL-6 after stimulation with *P*. *falciparum* CS protein [82]. Accordingly, higher IFN-γ and IL-6 production from control group PBMCs in this study suggested that innate cells, innate lymphoid cells and naive T-cells recognised lysate and produced cytokines [83].

Although stimulation with GBP 39113 had no differences cell proliferation regarding the control group, 46.6% of the exposed individuals’ cells proliferated following stimulus and the group’s mean remained above the cut-off line. An ideal anti-malarial vaccine candidate would be one which could activate T-cell (Th1 and Th2) response, inducing joint production of cytokines involved in cell proliferation and differentiation of innate and adaptive response, eliminating the parasite and promoting a boosted response by immune system memory cells. We thus propose GBP 39113 (having high binding affinity for HLA-DRβ1*04 and *11 allele lineages) and 39117 (having high binding affinity for HLA-DRβ1*07 and *13 allele lineages) as potential candidates for a vaccine against malaria caused by *P*. *vivax*.

Bioinformatics tools have proven to be quite useful in the process of vaccine development; predicting B- and T- cell epitopes *in silico* greatly narrows the amount of epitopes to be tested experimentally in *in vitro* antigenicity assays. Humoral and cellular immune responses against predicted B- and T-cell epitopes were here analysed in samples collected from individuals coming from an endemic region for *P*. *vivax* malaria. A similar approach has been followed in a previous study analysing *Pf*CelTOS protein comparing the *in silico* prediction of B- and T-cell epitopes with the *in vivo* immune response in animal models; such study suggested that optimized algorithms can be useful in vaccine design [26].

A bioinformatics approach combined with functional assays has allowed us to propose two protein regions containing both B- and T-cell epitopes within the *Pv*12 antigen that are able to induce a good immune response. 39038–39117 *Pv*12 region combines the best B- and T-cell epitopes and contains residues that could potentially interact with *Pv*41, standing out as a promising vaccine candidate, since it could trigger antibody/memory responses and block the binding activity to *Pv*41. The 39113–39040 region combines a good T-cell epitope, but a not so good B-cell epitope; however, it also contains potentially interacting residues with *Pv*41, and could be considered as another viable vaccine candidate.

Although the results described in the present study seem promising, caution must be applied considering previous data collected over the years. Several studies have shown that naturally-acquired immunity against *Plasmodium* infection is stage-, strain-, and species-specific. Studies immunising mice with either the C-terminal region of MSP-1 or AMA1 and then challenging with rodent Plasmodia, have shown only protective efficacy upon challenge with the homologous, but not a heterologous parasite strain [84–86]. A study in *Aotus* using three separate MSP1 fragments formulated in different adjuvants has shown a strain-specific response to challenge [87]. Another study immunising with recombinant AMA-1, assessed the response specificity and showed that AMA-1 was protective when challenging with a homologous *P*. *falciparum* strain. Nevertheless, AMA-1 was not protective when *Aotus* were challenged with a heterologous strain, suggesting again that the protective response induced by this protein is strain-specific [88]. Our group has invested great efforts in identifying those *P*. *falciparum* merozoite protein regions that are conserved (non-polymorphic between strains) and also participate in parasite binding to target cells (functionally relevant) by synthesising sequential 20-mer fragments of each, and testing their binding *in vitro*. Peptides displaying high binding have been named HABPs (High Activity Binding Peptides) [89–93]. After finding that conserved HABPs are not immunogenic, nor induce protection in the *Aotus* monkey model, it was decided to modify some amino acids which are critical in target cell binding [94], to allow a better fit into the HLA-DR molecules’ peptide-binding region [95,96].

Despite *Pv*12 peptides identified in the present study are antigenic, it is worth highlighting that most of them are conserved and, at least two of them, are functionally relevant (participating in binding to *Pv*41). Based on the observations made in *P*. *falciparum*, it is likely that modifications to *Pv*12 peptides to further improve their HLA-DR binding could be necessary before carrying out immunogenicity and protective efficacy assays in the *Aotus* monkeys, allowing the proper formation of the MHC-peptide-TCR complex, as has been shown earlier in *P*. *falciparum* modified HABPs [97].

## Supporting information

S1 Fig Antibody responses. **A. Immunoblot assay.** r*Pv*12 and r*Pv*GAMA protein recognition by exposed people’s sera. Positive control (anti-His) is shown in lane1, lanes 2 to 7 show the sera from 6 individuals living in endemic areas which recognised proteins as expected and lanes 8 and 9 show 2 sera from non-exposed volunteers. **B. Immunofluorescence assay.** a. One Tierralta exposed volunteer’s serum sample which recognised pRBC. b. One non-exposed volunteer’s (control group) serum sample. DAPI was used for staining nuclei, FITC-labelled anti-human IgG was used for staining the parasite and both were merged.
(PDF)

S2 Fig IgG antibody response. **(A) to r*Pv*GAMA as positive control**. Significant differences were observed between exposed individuals (n = 30) and control group (n = 8), (calculated by Mann-Whitney test). The dashed line indicates the cut-off point for seropositive samples. (***) *p*<0.0005. **B. IgG antibody response to *Pv*12 B-epitopes by endemic area.** Significant differences (calculated by Mann-Whitney test) are shown between samples from Colombia’s Chocó (n = 13) and Córdoba (n = 17) departments. The dashed line indicates the cut-off point for seropositive samples. (**) *p*<0.005.
(PDF)

S3 Fig Lymphocyte proliferative response to PHA. PBMCs from exposed and control groups were stimulated with PHA as positive control and Mann-Whitney was used for assessing statistically significant differences between exposed individuals and control group responses (*) *p*<0.05.
(PDF)

S1 File Interacting residues involved in P12 and P41 binding. The interacting positions, identity and similarity are shown in the sequences **A. *Pf*12 vs *Pv*12.** Primary sequence from *Pv*12, the *Pf*12-PDB (2YMO) crystal structure and *Pf*12 (C6KSX0) reference sequence. **B. *Pf*41 vs *Pv*41.**
(PDF)
