# Supplementary figures and images for: Plasmodium vivax Pv12 B-cell epitopes and HLA-DRβ1*-dependent T-cell epitopes in vitro antigenicity
Source: PLoS One. 2018 Sep 10;13(9):e0203715. doi: 10.1371/journal.pone.0203715 (PMC6130872; doi:10.1371/journal.pone.0203715)

**A.**

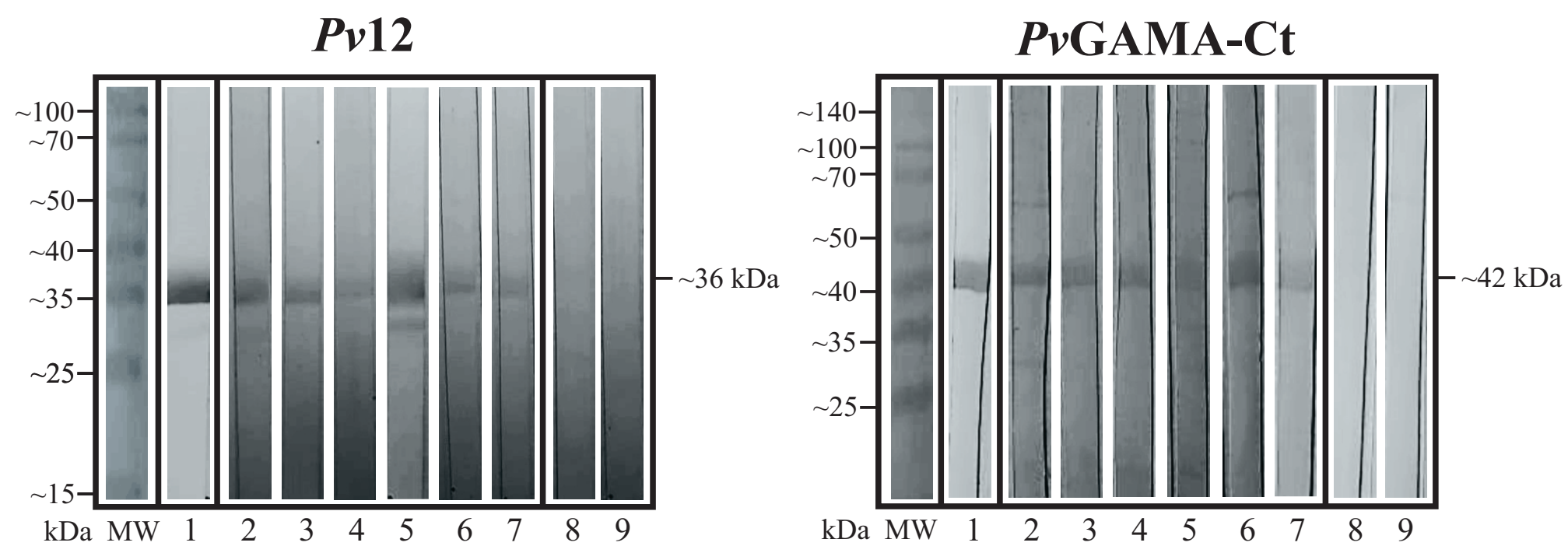

**B.**

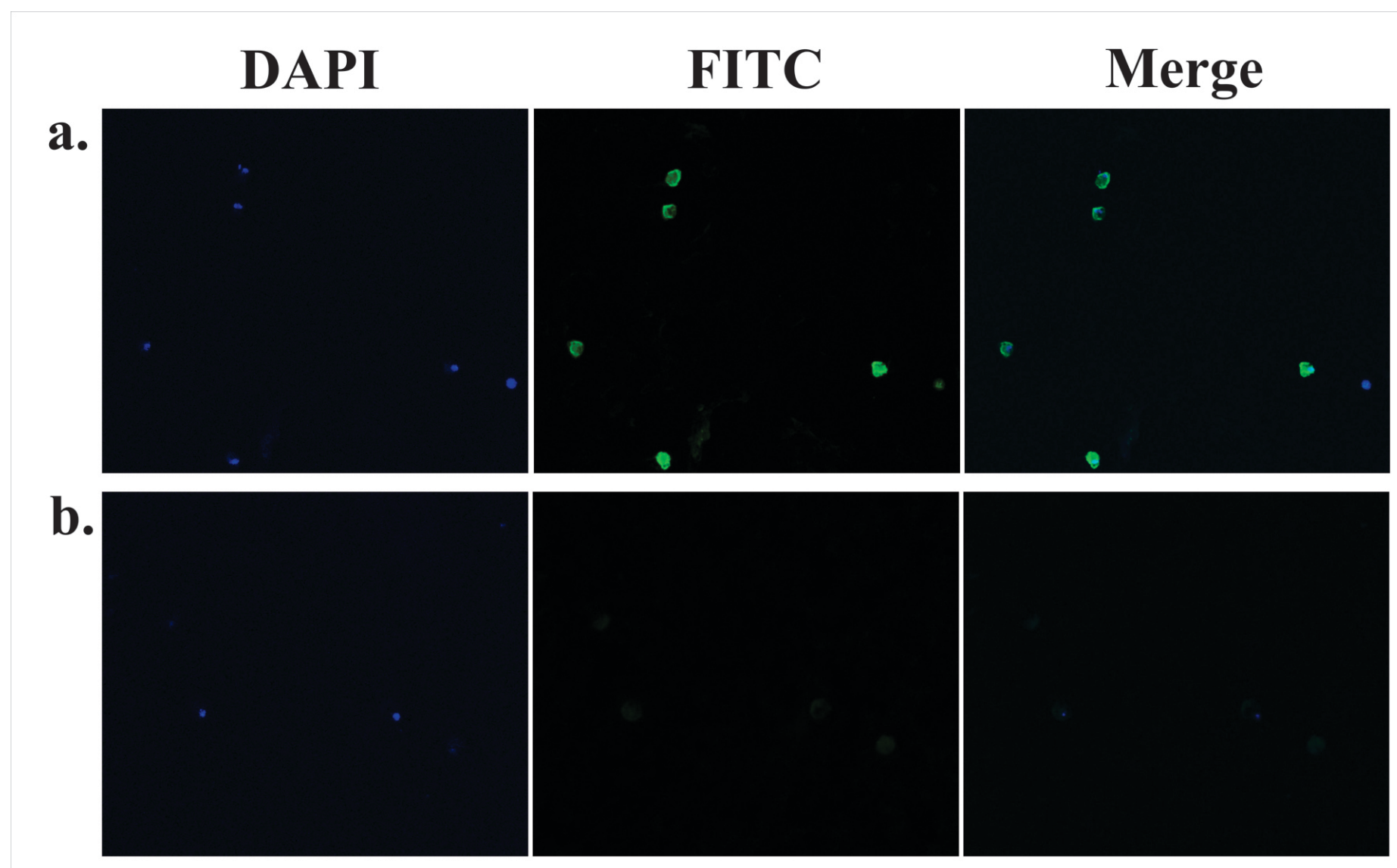

Supplement: S1 Fig — A. Immunoblot assay. rPv12 and rPvGAMA protein recognition by exposed people’s sera. Positive control (anti-His) is shown in lane1, lanes 2 to 7 show the sera from 6 individuals living in endemic areas which recognised proteins as expected and lanes 8 and 9 show 2 sera from non-exposed volunteers. B. Immunofluorescence assay. a. One Tierralta exposed volunteer’s serum sample which recognised pRBC. b. One non-exposed volunteer’s (control group) serum sample. DAPI was used for staining nuclei, FITC-labelled anti-human IgG was used for staining the parasite and both were merged. (PDF) [file pone.0203715.s001.pdf]

**A.**

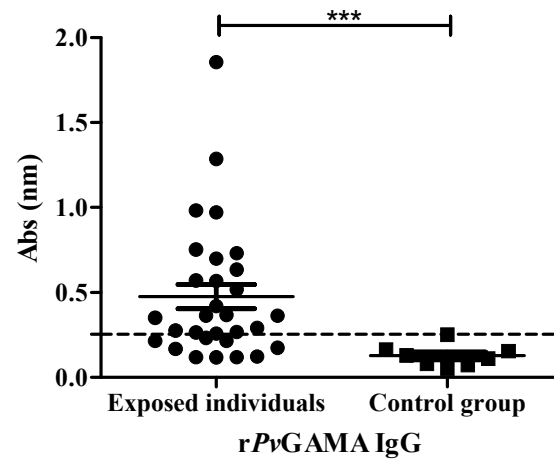

**B.**

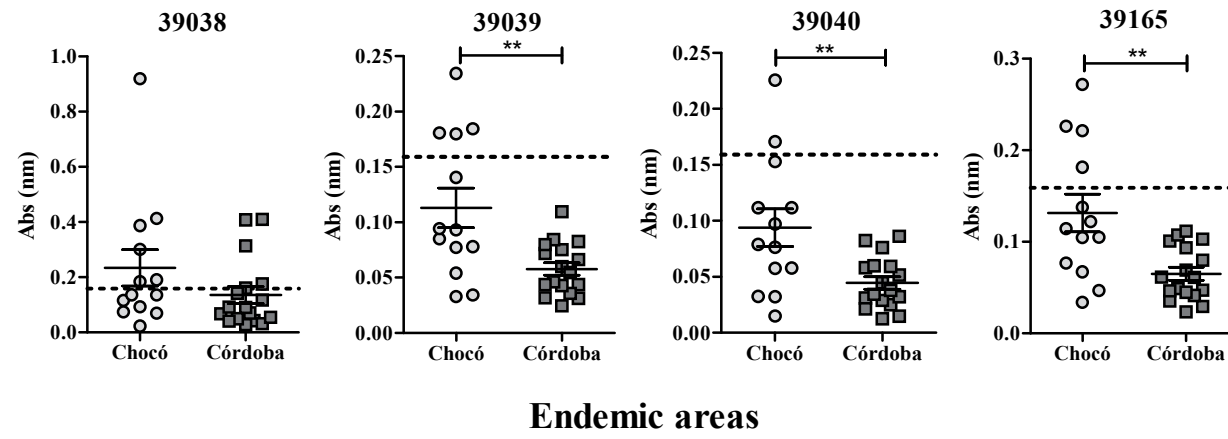

Supplement: S2 Fig — (A) to rPvGAMA as positive control. Significant differences were observed between exposed individuals (n = 30) and control group (n = 8), (calculated by Mann-Whitney test). The dashed line indicates the cut-off point for seropositive samples. (***) p<0.0005. B. IgG antibody response to Pv12 B-epitopes by endemic area. Significant differences (calculated by Mann-Whitney test) are shown between samples from Colombia’s Chocó (n = 13) and Córdoba (n = 17) departments. The dashed line indicates the cut-off point for seropositive samples. (**) p<0.005. (PDF) [file pone.0203715.s002.pdf]

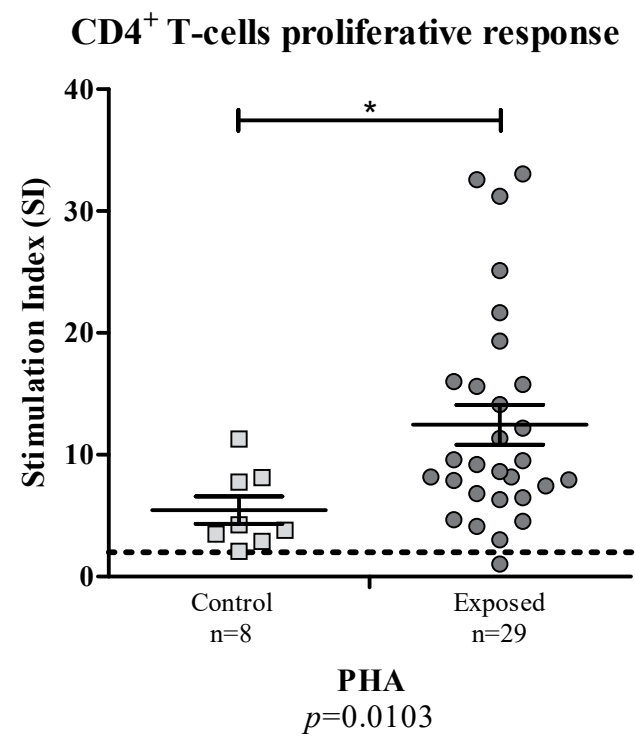

Supplement: S3 Fig — PBMCs from exposed and control groups were stimulated with PHA as positive control and Mann-Whitney was used for assessing statistically significant differences between exposed individuals and control group responses (*) p<0.05. (PDF) [file pone.0203715.s003.pdf]
