# Supplementary material for: Plasmodium vivax Pv12 B-cell epitopes and HLA-DRβ1*-dependent T-cell epitopes in vitro antigenicity
Source: PLoS One. 2018 Sep 10;13(9):e0203715. doi: 10.1371/journal.pone.0203715 (PMC6130872; doi:10.1371/journal.pone.0203715)
Supplement: S1 File — The interacting positions, identity and similarity are shown in the sequences A. Pf12 vs Pv12. Primary sequence from Pv12, the Pf12-PDB (2YMO) crystal structure and Pf12 (C6KSX0) reference sequence. B. Pf41 vs Pv41. (PDF) [file pone.0203715.s004.pdf]

Supplementary material 3. Interacting residues involved in P12 and P41 binding. The interacting positions, identity and similarity are shown in the sequences **A. *Pf*12 vs *Pv*12.** Primary sequence from *Pv*12, the *Pf*12-PDB (2YMO) crystal structure and *Pf*12 (C6KSX0) reference sequence. **B. *Pf*41 vs *Pv*41.**

A. Pf12 vs. Pv12

```
Pv12          M-RIAKAALCGQ-LLIWWL-SAPAEGFTHTCDFNDELSLEFDERQMTNGEQICTLKPDVL     57
Pf12-PDB      ----------------------------LTCDFNDVYKLEFHPNQQTSVTKLCNLTPNVL     32
Pf12-C6KSX0   MIKLSKKYCLGISFVLYILLSVCEGHKNLTCDFNDVYKLEFHPNQQTSVTKLCNLTPNVL     60
                                           ******  .***. .* *.  ::*.*.*:**

Pv12          DKVVIKCGAERKNYELLPNNCFEQVFTSKSIEDAKNAHNAHNVHNVQNAQQLVEYLHGAA     117
Pf12-PDB      EKVTIKCGSDKLNYNLYPPTCFE-------------------------------------     55
Pf12-C6KSX0   EKVTIKCGSDKLNYNLYPPTCFEEVYASRNMMHLK---------------KIKEFVIGSS     105
              :**.****::: **:* * .***

Pv12         AIVKRKQKETNADEKKSYDDVSFRVPPNLEKEQKALYCVCQNKAKIRVRKRSGEE-YDKE     176
Pf12-PDB     -----------------INEVSFRIPPNMMP-EKPIYCFCENK---------------KD     82
Pf12-C6KSX0  MFMRRSL------TPNKINEVSFRIPPNMMP-EKPIYCFCENKKTITINGSNGNPSSKKD     158
                               ::****:***:   :* :**.*:**               *:

Pv12         IFNLGIIEVLIPSLPKKIDGCDFTKNTSPLFTKGYDANFY--KTIENKDDVICKVKATEG     234
Pf12-PDB     IINRGIVEIIIPSLNEKVKGCDFTTSESTIFSKGYSINEIS-----NQQDIVCTVKAHAN     137
Pf12-C6KSX0  IINRGIVEIIIPSLNEKVKGCDFTTSESTIFSKGYSINEISNKSSNNQQDIVCTVKAHAN     218
             *:* **:*::**** :*:.*****.. * :*:***. *        *::*::*.***  .

Pv12         KLIGFKCPADYAIEPEECFLQGFNLSGKKEQLRTKIELTDLVMDHYNKIFYARVPQRIYQ     294
Pf12-PDB     DLIGFKCPSNYSVEPHDCFVSAFNLSGKNENL---LKLTNIIMDHYNNTFYSRLPSLISD     194
Pf12-C6KSX0  DLIGFKCPSNYSVEPHDCFVSAFNLSGKNENLENKLKLTNIIMDHYNNTFYSRLPSLISD     278
             .*******::*::**.:**:..******:*:*   ::**:::*****: **:*:*. * :

Pv12         NMHFFCACVLEEKRLVAH-FEFATTPDDQNSVSEPRAANVLQGRSAGSAAF---GPGPFL     350
Pf12-PDB     NWKFFCVCSKDNEKKLVFTVEASISSA---------------------------------     221
Pf12-C6KSX0  NWKFFCVCSKDNEKKLVFTVEASISSSNTKLAS--R-DNTYQDYISNSSFLTLSSYCAFI     335
             * :***.*  :::: :.. .* : :

Pv12         LLLLSGVLFFIM 362
Pf12-PDB     ------------ 221
Pf12-C6KSX0  TFIITSFLSFIL 347

Identity: 37%
Similarity (BLOSUM62): 56%

Interaction positions are highlighted in blue
```

B. Pf41 vs. Pv41

```
Pf41      MKGVI-FCLVVLLWRQAWVSSKSHKCDFTKEKYLLSGEKEVSCEIDANPSDDITFICPNK  59
Pv41      MKRLLLLALLHLVRQQLPAHAEEHICDFTKEKYLLGKNEKEYCVVNAKPFDSVTFICPKK  60
          ** :: :.*: *: :*  . ::.* **********. :::  * ::*:* *.:*****:*

Pf41      IDSLCFHTVNISKNINQN---KSTMSIQDLLYGSVVYGNTLFISPYVRTNTPFYCFCNLD  116
Pv41      IGAQCFQNVNTLDDISADKMESSKLSIDVLLYGSTLYGDTLLISPTVKQSTTFYCFCNLQ  120
          *.: **:.**  .:*. :   .*.:**: *****.:**:**:*** *: .* *******:

Pf41      TVTIQKFLKINRFLKDDDELSEADVMK--HLK------GGNVSEAQADEYLNKALNRFKK  168
Pv41      MEDLKKYLKKRRLTKEKENAKKKSTVNVNDLKNADEDMEVVVPEKQIDEHLVRALYRVKK  180
             ::*:** .*: *:.:: .: ..::  .**         * * * **:* :** *.**

Pf41      MKDLSKFFNDQADNTTKLNL--PKSLNIPNDILNYDVYNSSNNRNDIVVKDEVTNKQIIS  226
Pv41      IRNIIEREKNKGEGDKSTNQEDEEELVIEEE--------QEEEDGEGDEEDESKVEKIIT  232
          :::: :  :::.:. .. *    :.* * ::        ..:: .:   :** . ::**:

Pf41      KRGIMSVFVRSNNNVIKGCDFGNNNKNYFSHPISVAGKVNNKVCKIQGKPGELVGFKCAF  286
Pv41      KYGIMKVVVSTNNTITKGCDFGNNVVNYFSKPYPVERYGGSKVCRIEAKPGEFVGFKCIY  292
          * ***.*.* :**.: ********  ****:*  *    ..***:*:.****:***** :

Pf41      EENGKVEPPNCFDQVLHKNKVTDLKTLIPGYASYTNKHSSKYPYYLKIPHFVNEQYTIQC  346
Pv41      DNQGTVEPHNCFDKVFYEGKETDLQTLMPGYISYGNKQKGKYAFYLKLPHFVQHSYTVQC  352
          :::*.*** ****:*:::.* ***:**:*** ** **:..** :***:****:..**:**

Pf41      KCKSNNS-QNEYTFELDIQPGESEVVLNSFKTS   378
Pv41      KCRSTVHQYDDYVFELAVEGGESDIVAKSFQE-   384
          **:*.    ::*.*** :: ***::* :**:
```

Identity: 43%
Similarity (BLOSUM62): 60%

Interaction positions are highlighted in blue
